# Protein Amino Acid Composition: A Genomic Signature of Encephalization in Mammals

**DOI:** 10.1371/journal.pone.0027261

**Published:** 2011-11-23

**Authors:** Humberto Gutierrez, Atahualpa Castillo, Jimena Monzon, Araxi O. Urrutia

**Affiliations:** 1 Instituto de Fisiología Celular, Universidad Nacional Autónoma de México, Ciudad Universitaria, México City, México; 2 Department of Biology and Biochemistry, University of Bath, Bath, United Kingdom; American Museum of Natural History, United States of America

## Abstract

Large brains relative to body size represent an evolutionarily costly adaptation as they are metabolically expensive and demand substantial amounts of time to reach structural and functional maturity thereby exacerbating offspring mortality while delaying reproductive age. In spite of its cost and adaptive impact, no genomic features linked to brain evolution have been found. By conducting a genome-wide analysis in all 37 fully sequenced mammalian genomes, we show that encephalization is significantly correlated with overall protein amino acid composition. This correlation is not a by-product of changes in nucleotide content, lifespan, body size, absolute brain size or genome size; is independent of phylogenetic effects; and is not restricted to brain expressed genes. This is the first report of a relationship between this fundamental and complex trait and changes in protein *AA* usage, possibly reflecting the high selective demands imposed by the process of encephalization across mammalian lineages.

## Introduction

Encephalization, or increased brain size relative to body size, is highly variable in mammalian species [1]. This remarkable trait, however, entails substantial metabolic and developmental costs thereby imposing unique strains upon the entire pool of metabolic and energetic resources of the whole organism [2]–[6]. In spite of this, no features reflecting the genomic impact of brain evolution have been found so far. While a previous attempt to detect a genomic signature of brain evolution reported a widespread accelerated sequence evolution of genes functioning in the nervous system during human origins [7], this claim was heavily contested two years later [8].

By conducting a genome-wide analysis of amino acid composition in all 37 fully sequenced mammalian genomes, using multiple regression analysis, here we set out to investigate the impact of encephalization on protein amino acid composition, a genomic feature known to undergo evolutionary shifts in response to energy and metabolic pressures [9]–[13].

## Results

We adopted residuals of a log–log least squares linear regression of brain mass against body mass as the most appropriate index of encephalization (*Ei*). While direct estimates of the ratio of brain mass to body mass have also been used as an alternative encephalization index [3], [14], this measure, however, is known to be poorly related to brain complexity across taxa [15], [16]. We therefore used accurate estimates of brain residuals, based on a sample of 493 mammalian species, kindly provided by Gonzalez-Lagos [3]. Protein sequence data for the 37 fully sequenced mammalian species available to date were obtained from Ensembl [17]. Data and sources for brain mass, body mass, and other variables used throughout the study are presented as Table S1.

**Figure 1:**
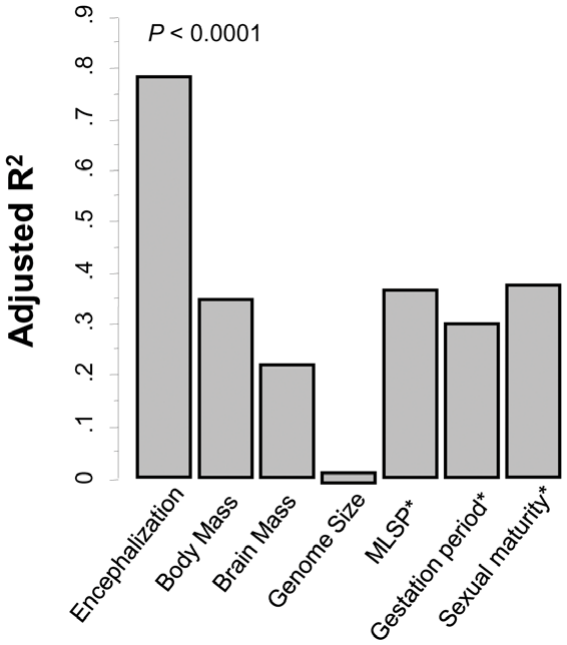
Overall protein *AA* frequencies correlate with encephalization. Chart showing *Adj.R^2^* values derived from multiple regressions including all 20 genomic *AA* frequencies per species as predictors of either encephalization index, brain mass, body mass, genome size, maximum life span (MLSP) gestational period or age of sexual maturity as predicted variables. Significance was numerically confirmed by a regression analyses against 10, 000 permutations of each of the above dependent variables. * Log-transformed values.

Direct simple regressions between genome-wide averages for individual *AA* frequencies in all 37 species and *Ei*, shows no significant correlation for any individual *AA* after Bonferroni correction (Table S2). However, multiple regression analysis including all 20 *AA*s as predictors of encephalization, where significance was assessed by 10000 random permutations of *Ei* values, revealed, despite the large number of predictors included, a strong association between amino acid composition and encephalization (*Adj. R^2^* = 0.785, *P*<0.0001; Figure 1).

Because *Ei* is a function of both absolute brain and body mass, the observed association could be secondary to underlying relationships with either variable. However, neither brain mass nor body mass were significantly related with whole genome average *AA* frequencies (*P*>>0.001; Figure 1), suggesting a specific association between protein *AA* usage and encephalization. Only comparisons against absolute brain and body mass are shown in Figure 1 as log-transformed values of these two variables and *Ei* are known to remain significantly correlated with each other [3]. Even so, neither log [brain mass] nor log [body mass] were significantly related with whole genome average *AA* frequencies (*Adj. R^2^* = 0.625 and 0.578 respectively, *P*>0.001 in both cases).

Reductions in effective population size, which are known to correlate with increased body size [18], could potentially alter *AA* composition due to relaxed selection for optimal genome-wide *AA* usage. The lack of correlation, however, between either body size or genome size – another indicator of increased genetic drift [18]- with *AA* frequencies across species, indicates that the observed pattern is not the result of reduced effective population size (Figure 1).

Comparative work on brain evolution has uncovered a robust relationship between relative brain size and lifespan [3], [19], [20]. However, the observed correlation between *AA* usage and *Ei* is not secondary to an underlying link between protein composition and lifespan as no significant association between overall *AA* usage and either maximum lifespan (MLSP), gestation period, or age of sexual maturity was found (Figure 1).

Essentially the same results were obtained when restricting the analyses to only 1779 sets of ortholog genes covering all 37 species, demonstrating, in spite of the reduced sample, that the observed pattern is not the result of distorted average *AA* frequencies due to uneven gene duplication events across species (Figure S1).

Lastly, the observed association between *AA* content and encephalization is not the spurious result of underlying shifts in DNA nucleotide composition as no significant correlation was observed between *Ei* and proportion of G+C nucleotides per gene averaged across all protein-encoding sequences per species (*R^2^* = 0.052; *P* = 0.191, Figure S2). In addition, no correlation was detected between the proportion of body mass that corresponds to brain mass (brain to body mass ratio) and overall protein *AA* usage (data not shown), indicating that the associative pattern we observe is specifically restricted to degree of encephalization when this trait is expressed as the excess of brain mass not accounted for by the allometric increase in body size.

**Figure 2:**
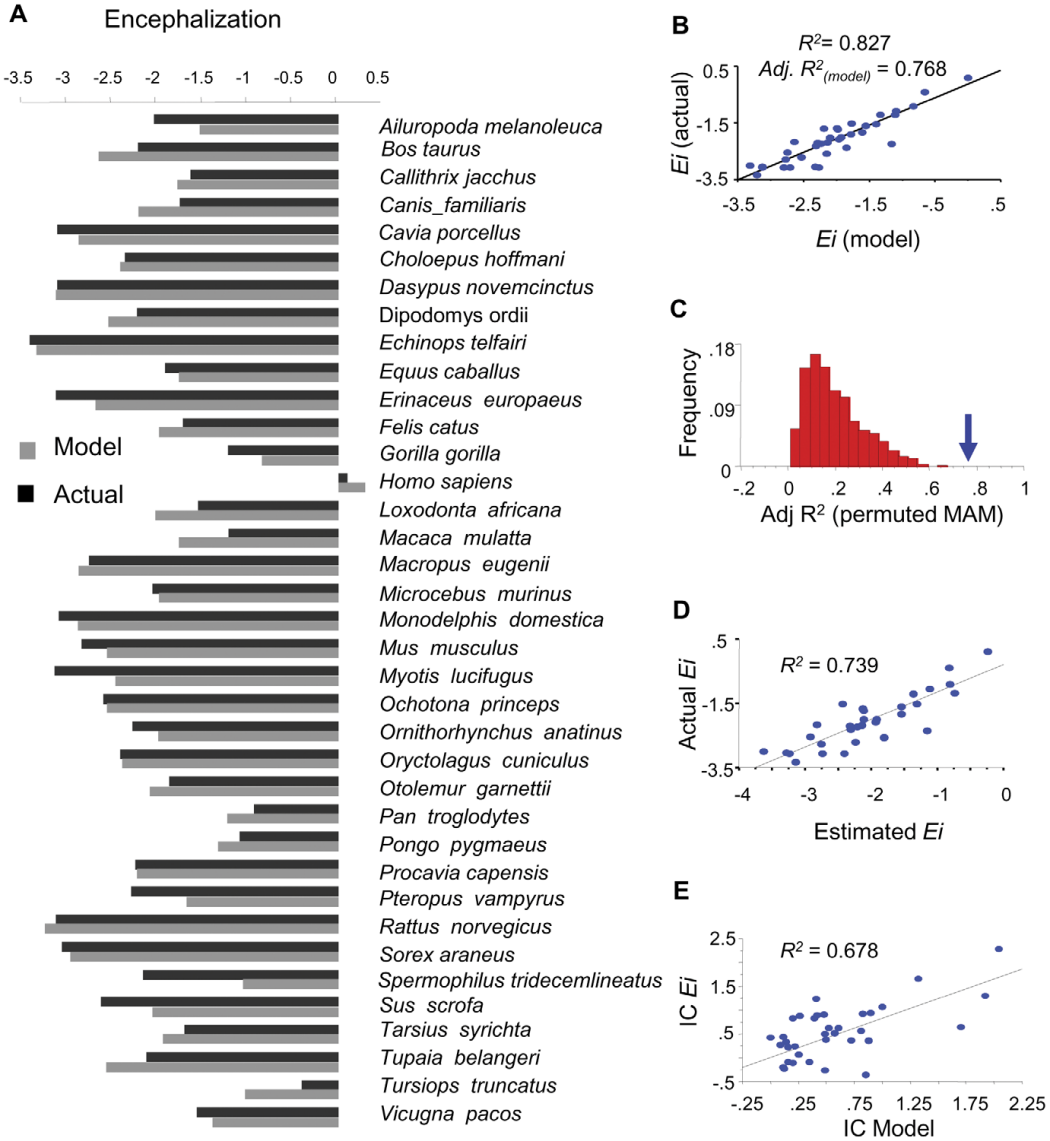
Performance of optimal multiple regression model predicting encephalization. The minimum adequate model (MAM) with the highest *F* statistics was exhaustively searched for within the corresponding space of 2^20^ possible models (see Table 1). A) Bar chart showing comparisons between actual *Ei* values per species (black bars) and the values derived from the corresponding optimal model based on *AA* composition (grey bars). B) Graph showing in-sample goodness of fit between actual and *AA*-based estimates of *Ei* values. *R* ^2^, and *Adj.R^2^* values are shown . C) Distribution of *Adj.R^2^* values corresponding to control MAMs obtained for 1000 independent permutations of *Ei* Values. The blue arrow indicates the value of the actual model in panel B (*P*<0.001). D) Over-fitting control analysis showing the performance of the optimal linear model predicting encephalization. All species were randomly sorted into nine groups and the *Ei* values of each group were predicted using coefficients derived *de novo* from the remaining out-of- group species. The graph shows the relationship between the actual and predicted values with the corresponding *R^2^* and associated *P* value shown. E) Independent contrast analysis, which controls for phylogenetic effects, was carried out between *Ei*, and the *AA* usage-based estimates of *Ei*. The relevant phylogeny for all 37 species was obtained from Ensembl genome data resource and standardized contrasts (IC) were generated using the PDAP: PD TREE module in the Mesquite environment. (*R^2^* = 0.678, through the origin, *P*<0.0001).

Because our independent variables are expressed in terms of frequencies, co-linearities and redundancies between *AAs* are bound to occur. We therefore tested the strength of the observed pattern, by generating a minimum adequate model (MAM) capable of estimating the level of encephalization solely as a function of a minimum set of genome-wide *AA* frequencies. The resulting MAM predicting encephalization showed a considerably high in-sample performance (*R^2^* = 0.825; Table 1 and Figure 2A and B) which starkly contrasts with the poorly performing MAM predicting a random permutation of *Ei* values (*R^2^* = 0.244, Figure S3). To properly assess the statistical significance of this result we extracted MAMs for 1000 random permutations of *Ei* values and the resulting distribution of *Adj. R^2^* values shows that the model predicting the real *Ei* values performs significantly better than expected by chance (*P*<0.001, Figure 2C).

**Table 1:** Minimum adequate model for *Ei*, *F* = 13.841; *R_2_* = 0.827, *adj. R_2_ = *0.768.

	Coefficient	t-Value	P-Value
Intercept	−372.23	−5.796	<.0001
A	523.352	3.367	0.0024
D	−863.086	−5.912	<.0001
E	1431.306	7.701	<.0001
G	316.765	2.516	0.0184
I	1167.566	5.211	<.0001
P	441.401	3.492	0.0017
S	1300.219	4.91	<.0001
W	1098.496	2.846	0.0085
V	964.661	6.162	<.0001

Predictor amino acids are expressed in single letter code.

Because multiple regressions frequently risk over-fitting (i.e, only able to predict the same data used to generate them), we randomly sorted all species into nine groups and the corresponding *Ei* values of each group were predicted using linear coefficients derived *de novo* from the remaining out-of-group species using the same predictors of Table 1. Figure 2D shows a statistically significant fit between out-of-sample estimations and actual *Ei* values, demonstrating that overall *AA* usage can actually explain over 70% encephalization variance among mammals.

In order to eliminate phylogenetic contributions to the observed correlation between encephalization and *AA* usage we carried out an independent contrast analysis (IC) [21]–[23] between *Ei*, and the *AA-*based estimates of *Ei*. This analysis tests the observed association under the assumption of maximum phylogenetic correlation between variables. The relevant phylogeny was obtained from Ensembl [17] and standardized ICs were generated using the PDAP:PD TREE [22] module in the Mesquite program [24]. The significant correlation between IC from *AA*-based estimates and actual *Ei* values, demonstrates that the observed association between encephalization and *AA* usage holds even assuming maximum phylogenetic autocorrelation (Figure 2E; *R^2^* = 0.678, *P*<0.0001). In order to test whether phylogenetic effects are actually present in our *Ei* data, we used a phylogenetic generalized least square approach (PGLS) to determine the parameter λ, which measures the degree to which the phylogeny predicts the pattern of covariance among species (where λ values close to 0 represent no phylogenetic autocorrelation while values close to 1 represent full phylogenetic autocorrelation) [23], [25]. Maximum likelihood estimation of the λ parameter revealed an intermediate level of phylogenetic autocorrelation for *Ei* (λ = 0.788; *P* = 0.005 that λ = 0; *P*<0.0001 that λ = 1), indicating that the actual phylogenetic effects are weaker than those assumed by the independent contrasts analysis. Indeed, the fitted PGLS model for *Ei*, using the same predictor amino acids of Table 1, revealed a much stronger association than the one obtained with independent contrasts (PGLS : F_(10,27)_ = 11.05, *Adj. R^2^* = 0.715, P<0.0001). Taken together, these results demonstrate that the observed association between encephalization and protein *AA* usage holds independently of phylogenetic relationships.

**Figure 3:**
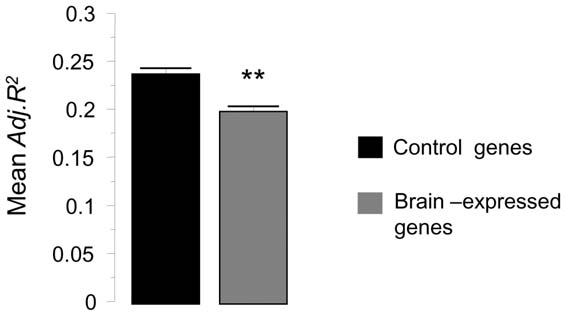
*AA* usage and encephalization correlation is not restricted to brain-expressed genes. We compiled 12456 sets of orthologous genes encompassing all 37 species, and each set containing at least 20 orthologs. For each sets, we obtained the multiple regression coefficients (*Adj. R^2^*) between *AA* frequencies (per gene per individual set of orthologs) and *Ei* values of all 37 species using the same predictors of Table. We then obtained human tissue expression data from BodyMap database for 18 tissues with known expression in over 50% of all human genes also present in our database. We identified 10647 brain-expressed genes and 1594 genes with known expression specifically outside the brain (control genes). Bars in the graph compare the mean *Adj. R^2^*value of these control genes (±SEM) with the corresponding mean value of brain-expressed genes. Confidence interval for brain-expressed genes, was estimated using100 equally sized random samples. ** P<0.0001.

Given the robust link between protein composition and encephalization, we asked whether this correlation was particularly pronounced in brain-expressed genes. To this end, we compiled a database of 12459 sets of orthologous genes relative to human, and each set was required to contain orthologs from at least 20 of the 37 species. For each one of the 12459 sets of orthologs, we obtained the multiple regression coefficient (*Adj. R^2^*) between *AA* frequencies (per gene per set of orthologs) and *Ei* values of all 37 species using the same predictors of Table 1. We obtained human tissue expression data from BodyMap database [26], for 18 different tissues including brain and identified genes with known expression in the brain and genes expressed specifically outside the brain (control genes). By comparing the mean *Adj. R* value of control genes with that of 100 equally-sized random samples of brain-expressed genes, we surprisingly found a significantly lower average correlation in brain-expressed genes (Figure 3), showing that correlated genes are certainly not preferentially enriched among brain-expressed genes, and suggesting that highly correlated genes can be specifically expressed anywhere outside the brain.

## Discussion

In this study, we have demonstrated a significant correlation between genome-wide patterns of protein *AA* usage and degree of encephalization in mammalian species.

While the direction of the cause-effect relationship cannot be established with certainty due to the correlational nature of the study, there is little doubt that the high metabolic cost of the mammalian brain entails unique pressures upon the entire pool of metabolic and energetic resources affecting the entire organism [3]–[6]. Regarding its impact on protein composition, this cost could be realized in three general ways: 1) Metabolic cost of amino acid synthesis has been linked to patterns of protein composition across several taxa [9]–[13] overall, suggesting that organisms adapt their protein composition to their energetic budgets. The uniquely high energetic requirements for normal brain function could conceivably impose systemic energy shortages to which organisms may adapt by shifting toward metabolically cheaper protein compositions. Detailed studies of acquisition or production costs of individual amino acids in mammals linked to studies of the relative proportion of metabolic energy allocated to the brain will shed light on the potential link between brain-related energy demands and overall protein *AA* usage. 2) Dietary changes are known to have accompanied increased encephalization in birds and mammals [5], [6], [27], [28]. While acting as a critical co-adjuvant factor, diet could also act as a metabolic restraint limiting availability of critical *AA*s or precursors in favor of energy rich nutrients. Future studies on diet–related availability of amino acids linked to encephalization will help to clarify the potential link between brain-related dietary shifts and protein *AA* usage. 3) Alternatively, but much less investigated, is the potential impact of systemic changes in the relative abundance of certain key *AA* resulting from high brain-related demands of a number of metabolites destined to neural-specific functions not related to protein synthesis (i.e., neurotransmitters and neuromodulators). The resulting shortages in the systemic availability of some amino acids could in turn lead to adaptive compositional shifts affecting genes expressed anywhere in the organism.

While the observed shifts seem to affect several amino acids, this fact should be interpreted with caution as many of the observed biases could still be the result of selective changes on a much reduced set of individual *AA*s that could in turn spread via nonlinear compensatory changes to other amino acid residues leading to additional significant shifts in frequency.

Taken together our results demonstrate a robust association between genome-wide patterns of protein *AA* usage and encephalization in mammals. Furthermore, they demonstrate that this association is not secondary to body mass, brain size, lifespan or nucleotide content. Because this correlation seems not to be restricted to proteins specifically present in the nervous system, our results suggest that, throughout evolution, mammalian proteins have adjusted their composition in response to general energy and/or metabolic pressures brought about by increases of relative brain size.

## Methods

We adopted residuals of a log–log least squares linear regression of brain mass against body mass as an index of encephalization (*Ei*). Accurate estimates of grade shift-corrected brain residuals based on a sample of 493 mammalian species were kindly provided by Gonzalez-Lagos [3]. We used protein sequence data for the 37 fully sequenced mammalian species available to date from Ensembl [17] and genome-wide averages for individual *AA* frequencies per species were obtained for subsequent statistical analysis. Data and sources for brain mass, body mass, and other variables used throughout the study are presented as Table S1.

Genes with orthologs relative to human present in all 37 species were identified using Ensembl orthology data. Where multiple orthologs were identified in a given species, one was randomly chosen. From the resulting set of 1779 genes, average frequencies for each amino acid were obtained per species. For the analysis shown in figure 3, 12459 sets of orthologous genes relative to human, were obtained in the same way with the difference that each set was required to contain orthologs in at least 20 of the 37 species. Human tissue expression data was obtained from BodyMap database [26].

For the Independent Contrast analysis, the relevant phylogeny was obtained from Ensembl [17] and standardized ICs were generated using the PDAP:PD TREE [22] module in the Mesquite program [24]. Branch lengths in the phylogenetic three were adjusted using Nee's method [5]. PGLS and maximum likelihood estimation of λ values were carried out using the R's-based Caper package. Simple Pearson correlations and multiple regressions were carried out using R and Matlab-based statistical functions. Numerical randomizations were conducted using specially written Matlab and R-based scripts.

## Supporting Information

Figure S1 **Multiple regression analysis carried out in sets of orthologous genes.** Genes with orthologs relative to human present in all 37 species were identified using Ensembl orthology data. Where multiple orthologs were identified in a given species, one was randomly chosen. From the resulting set of 1779 genes, average frequencies for each amino acid were obtained per species. Chart shows *Adj.R^2^* values derived from multiple regressions including all 20 *AA* frequencies per species as predictors of either encephalization index, brain mass, body mass, genome size, maximum life span (MLSP) , gestational period or age of sexual maturity as predicted variables. Significance was numerically confirmed by a regression analyses against 10, 000 permutations of each of the above dependent variables. * Log-transformed values.
(PDF)

Figure S2 **Lack of correlation between DNA nucleotide content and encephalization.** Graph showing linear regression between *Ei* and mean percentage G+C content of protein-encoding sequences (including introns) per species. G+C contents for all species were obtained from Ensemble data resources. (*P*>0.1).
(PDF)

Figure S3 **Performance of minimum adequate model (MAM) using mean amino acid frequencies per species as predictors and a random permutation of** ***Ei*** **values as the predicted variable.** X axis corresponds to the model-based prediction of the permuted *Ei* value. Note the lack of significance of the resulting adjusted *R^2^* coefficient relative to the performance distribution of MAMs obtained for 1000 control permutations of *Ei* values (Figure 2C of the main manuscript).
(PDF)

Table S1 **Data and sources for variables used throughout the study.**
(PDF)

Table S2 **Correlation coefficient (R) values and associated probabilities (P) obtained after linear correlations comparing mean genomic** ***AA*** **frequencies and encephalization index.**
(PDF)
