# Supplementary figures and images for: Protein Amino Acid Composition: A Genomic Signature of Encephalization in Mammals
Source: PLoS One. 2011 Nov 23;6(11):e27261. doi: 10.1371/journal.pone.0027261 (PMC3223171; doi:10.1371/journal.pone.0027261)

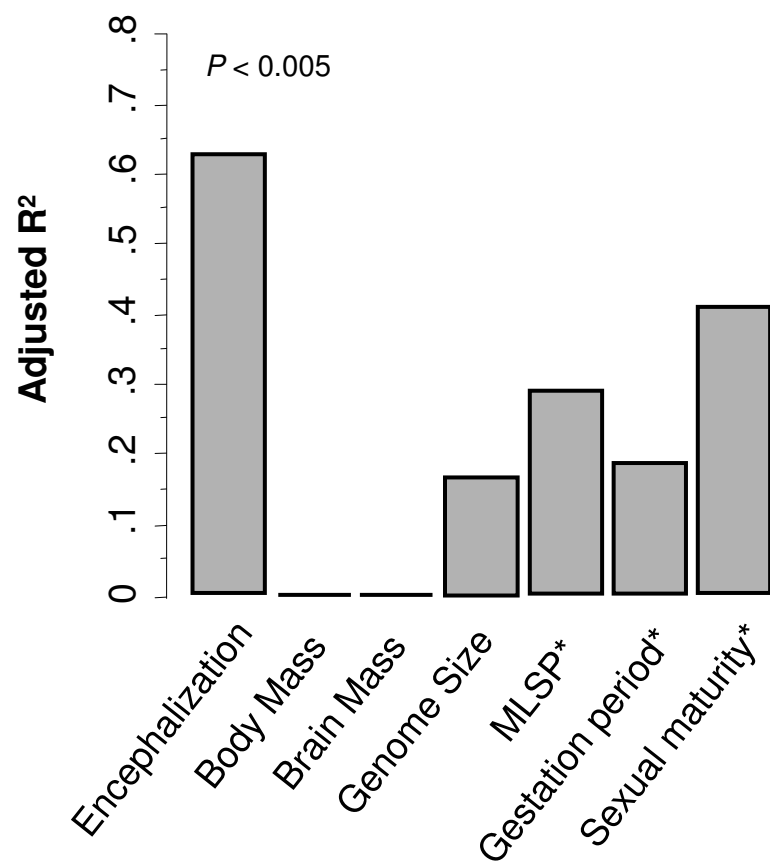

Supplement: Figure S1 — Multiple regression analysis carried out in sets of orthologous genes. Genes with orthologs relative to human present in all 37 species were identified using Ensembl orthology data. Where multiple orthologs were identified in a given species, one was randomly chosen. From the resulting set of 1779 genes, average frequencies for each amino acid were obtained per species. Chart shows Adj.R2 values derived from multiple regressions including all 20 AA frequencies per species as predictors of either encephalization index, brain mass, body mass, genome size, maximum life span (MLSP) , gestational period or age of sexual maturity as predicted variables. Significance was numerically confirmed by a regression analyses against 10, 000 permutations of each of the above dependent variables. * Log-transformed values. (PDF) [file pone.0027261.s001.pdf]

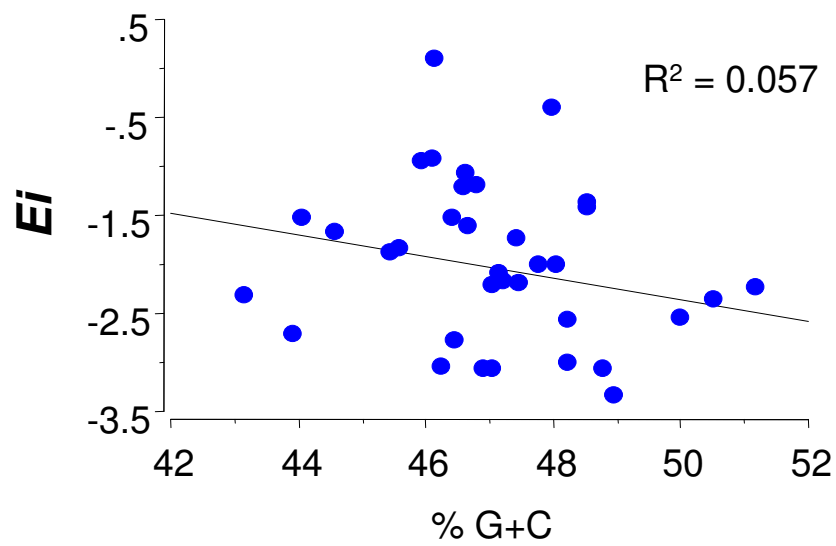

Supplement: Figure S2 — Lack of correlation between DNA nucleotide content and encephalization. Graph showing linear regression between Ei and mean percentage G+C content of protein-encoding sequences (including introns) per species. G+C contents for all species were obtained from Ensemble data resources. (P>0.1). (PDF) [file pone.0027261.s002.pdf]

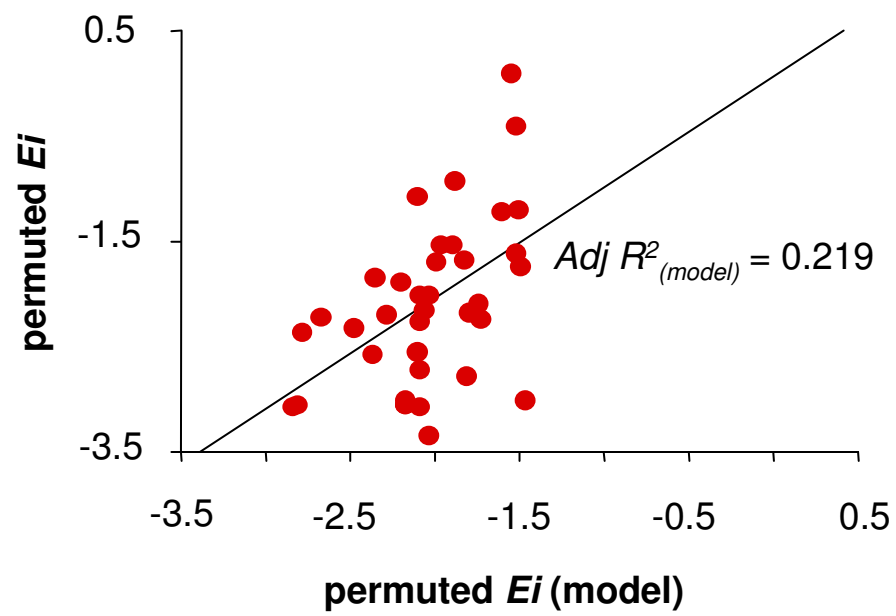

Supplement: Figure S3 — Performance of minimum adequate model (MAM) using mean amino acid frequencies per species as predictors and a random permutation of Ei values as the predicted variable. X axis corresponds to the model-based prediction of the permuted Ei value. Note the lack of significance of the resulting adjusted R2 coefficient relative to the performance distribution of MAMs obtained for 1000 control permutations of Ei values (Figure 2C of the main manuscript). (PDF) [file pone.0027261.s003.pdf]
