# Supplementary material for: Protein Amino Acid Composition: A Genomic Signature of Encephalization in Mammals
Source: PLoS One. 2011 Nov 23;6(11):e27261. doi: 10.1371/journal.pone.0027261 (PMC3223171; doi:10.1371/journal.pone.0027261)
Supplement: Table S1 — Data and sources for variables used throughout the study. (PDF) [file pone.0027261.s004.pdf]

Table S1. Data and sources for variables used throughout the study

Species	MLSP[1]	Sexual Maturity[1]	Gestation[1]	Brain Mass[3-7]	Body Mass[3-7]	Genome size[2]	*EI*[8]
Ailuropoda melanoleuca	36.8	2192	48	235.1	117920	2299.49221	-2
Bos taurus	20	548	277	456	520000	2918.20564	-2.17
Callithrix jacchus	16.5	429.5	144	7.24	280	2933.98265	-1.6
Canis familiaris	24	510	63	100.9	19240	2531.67395	-1.72
Cavia porcellus	12	71	68	4.28	971	2723.21964	-3.04
Choloepus hoffmani	37	912.5	350	28.5	4000	2467.49319	-2.31
Dasypus novemcinctus	22.3	365	133	12	3700	4824.20113	-3.04
Dipodomys ordii	9.9	61	29	1.97	54	2165.2948	-2.18
Echinops telfairi	19	365	55	0.52	60	3833.56839	-3.34
Equus caballus	57	943.5	337	650.03	441175	2474.9124	-1.88
Erinaceus europaeus	11.7	253	30	3.77	697	3377.52412	-3.06
Felis catus	30	289	65	28.4	2500	4055.84759	-1.68
Gorilla gorilla	55.4	3422	256	438.18	122500	3041.97616	-1.2
Homo sapiens	122.5	4927.5	280	1300	65000	3101.80474	0.1
Loxodonta africana	65	3834	670	4480	2750000	3196.74397	-1.52
Macaca mulatta	40	1619	165	97.45	8250	3097.17996	-1.19
Macropus eugenii	15.1	502	28	23.7	4425	2955.77394	-2.7
Microcebus murinus	18.2	243	61	1.68	50	2910.10301	-2.01
Monodelphis domestica	5.1	122	15	0.95	100	3605.63173	-3.03
Mus musculus	4	42	19	0.45	24	2716.96548	-2.78
Myotis lucifugus	34	210	55	0.175	8	2858.7162	-3.07
Ochotona princeps	7	347	30	2.39	169	4781.88732	-2.54
Ornithorhynchus anatinus	22.6	548	17	9.22	1030.3	2073.14863	-2.23
Oryctolagus cuniculus	9	730	30	9.14	1411.8	2737.47326	-2.36
Otolemur garnettii	18.3	600	132	10.45	946.7	3429.34303	-1.83
Pan troglodytes	59.4	3148	229	371.05	45500	3350.41334	-0.91
Pongo pygmaeus	59	2555	249	343	36900	3446.7714	-1.07
Procavia capensis	14.8	500	215	20.5	3800	2993.32306	-2.2
Pteropus vampyrus	20.9.	.		9.53	1060	1999.61446	-2.24
Rattus norvegicus	5	80	21	2.38	339	2718.89733	-3.06
Sorex araneus	3.2	266.5	21	0.23	8.4	2944.19305	-2.99
Spermophilus tridecemlineatus	7.9	289.5	28	3.2	175	3499.04961	-2.12
Sus scrofa	27	551	115	180.2	158320	2262.59641	-2.57
Tarsius syrichta	16.		179	3.5	117	3187.36614	-1.67
Tupaia belangeri	11.1.		46	3.1	150	3670.32464	-2.08
Tursiops truncatus	51.6	3260	365	1679.6	180910	2521.90755	-0.4
Vicugna pacos	25.8	466	345	188	50000	2967.72948	-1.53

1) AnAge database (http://genomics.senescence.info/species/). **2)** Ensemble data sources http://www.enselmb.org. **3)** Dunbar, R. I. & Shultz, S. Understanding primate brain evolution. *Philos Trans R Soc Lond B Biol Sci* 362, 649-658 (2007). **4)** McNab, B. K. & Eisenberg, J. F. Brain Size and Its Relation to the Rate of Metabolism in Mammals. *The American Naturalist* 133, 157-167 (1989). **5)** Leonard, W. R., Snodgrass, J. J. & Robertson, M. L. Effects of brain evolution on human nutrition and metabolism. *Annu Rev Nutr* 27, 311-327 (2007). **6)** Gittleman, J. L. Carnivore Brain Size, Behavioral Ecology, and Phylogeny. *Journal of Mammalogy* 67, 23-36 (1986). **7)** Stephan, H., Nelson, J. E. & Frahm, H. D. Brain size comparison in Chiroptera. *Journal of Zoological Systematics and Evolutionary Research* 19, 195-222 (1981). **8)** Grade shift-corrected brain residuals from Gonzalez-Lagos, C., Sol, D. & Reader, S. M. Large-brained mammals live longer. *J Evol Biol* 23, 1064-1074, (2010).
