# Supplementary material for: Protein Amino Acid Composition: A Genomic Signature of Encephalization in Mammals
Source: PLoS One. 2011 Nov 23;6(11):e27261. doi: 10.1371/journal.pone.0027261 (PMC3223171; doi:10.1371/journal.pone.0027261)
Supplement: Table S2 — Correlation coefficient (R) values and associated probabilities (P) obtained after linear correlations comparing mean genomic AA frequencies and encephalization index. (PDF) [file pone.0027261.s005.pdf]

Table S2. Correlation coefficient (R) values and associated probabilities (P) obtained after linear correlations comparing mean genomic *AA* frequencies and encephalization index.

Amino Acid	***R* value**	***P* values**
A	0.165	0.3696
R	0.287	0.1115
N	-0.207	0.2589
D	-0.345	0.0529
C	0.104	0.573
E	0.196	0.2848
Q	0.118	0.5242
G	0.334	0.0617
H	0.244	0.1793
I	-0.24	0.1867
L	-0.324	0.0703
K	-0.062	0.739
M	0.009	0.9616
F	-0.304	0.0904
P	0.413	0.0182
S	0.009	0.9605
T	-0.247	0.1735
W	0.475	0.0054
Y	-0.471	0.0059
V	-0.429	0.0135

Amino Acids are expressed in single letter code
